# Decoding How Articulation and Pauses Influence Pronunciation Proficiency in Korean Learners of English

**DOI:** 10.3390/bs16020179

**Published:** 2026-01-26

**Authors:** Tae-Jin Yoon, Seunghee Han, Seunghee Ha

**Affiliations:** 1 Department of English Language and Literature, Sungshin Women’s University, Seoul 02844, Republic of Korea; 2 Big Data Convergence & Open Sharing System (COSS), Seoul National University, Seoul 08826, Republic of Korea; 3 Division of Speech Pathology and Audiology, Research Institute of Audiology and Speech Pathology, Hallym University, Chuncheon 24252, Republic of Korea

**Keywords:** rater’s bias, pronunciation proficiency, articulation rate, mean silence mean, script complexity, Korean learners of English

## Abstract

This study investigates how temporal fluency cues shape human ratings of L2 English pronunciation in Korean learners, using a large read-speech corpus annotated with five-point pronunciation scores. We focus on two timing-derived measures—articulation rate (AR) and mean silence duration (SilMean)—and examine whether these cues predict (i) articulation-accuracy ratings and (ii) prosody/fluency ratings. To account for dependencies in corpus data and to control for key learner- and task-level covariates, we fitted cumulative link mixed models with random intercepts for speakers and scripts, including proficiency band (ability), age, gender, and test type as fixed effects. Across models, faster articulation and shorter silent intervals were associated with higher articulation ratings, and a combined model including both AR and SilMean provided the best fit (lowest AIC). Temporal cues were even more strongly associated with prosody ratings, supporting construct alignment between timing measures and the prosody dimension of the rubric. Marginal predicted probabilities illustrate how the likelihood of receiving high ratings (score ≥ 4) increases with AR across proficiency and linguistic-complexity strata (with SilMean held constant), and how long silent intervals reduce these probabilities when AR is held constant. These findings indicate that temporal organization provides robust information about perceived pronunciation quality in read L2 speech and underscore the importance of construct-aware modeling when developing AI-based scoring and feedback systems trained on human-labeled data.

## 1. Introduction

The integration of Artificial Intelligence (AI) into English language learning has become increasingly significant, particularly in enhancing core communication skills such as speaking and writing (Evanini & Zechner, 2019; Gong, 2023). AI technologies are now capable of interpreting and translating language (Zhu & Guan, 2020), providing learners with immediate feedback and personalized instruction. A critical component of effective AI-assisted language learning is the utilization of expert-labeled data in supervised learning, which has been shown to substantially improve AI performance (Chawla & Karakoulas, 2005; Mitchell, 1997; Yang & Yu, 2020). In the context of pronunciation and speaking education for learners of English as a foreign language, precise evaluative feedback from AI systems is contingent upon meticulously labeled pronunciation data (Latif & Zhai, 2024; Settles et al., 2020). Accurate proficiency scoring by AI not only enhances the learning experience but also allows educators to allocate resources more efficiently, focusing on other essential aspects of language development.

Despite these advancements, current AI language models like ChatGPT face limitations in assessing fluency in spoken English. While models such as ChatGPT excel in generating coherent and contextually appropriate text, they are primarily designed for text-based interactions and lack the capability to process acoustic signals inherent in spoken language. This means they cannot directly analyze essential components of speech such as pronunciation, intonation, stress, and rhythm, which are crucial for evaluating fluency (Chen et al., 2024). Consequently, these models may not accurately capture the nuances of non-native speech patterns, including accents and speech disfluencies, limiting their effectiveness in providing comprehensive fluency assessments. This limitation highlights the need for specialized AI systems that can process and evaluate spoken language by integrating both linguistic and acoustic analyses (Evanini & Zechner, 2019; Latif & Zhai, 2024).

To address the need for high-quality data in developing such specialized AI systems, we utilized a speech corpus constructed through a project funded by the National Information Society Agency (NIA) of Korea. This initiative, part of NIA’s support program for building AI training data, aimed to create a substantial dataset necessary for the development of AI-assisted speech communication systems. The corpus comprises 400 h of speech from 882 Korean learners of English, providing a rich resource for analyzing pronunciation proficiency through both acoustic-prosodic fluency-related features. Although the corpus was constructed with substantial financial resources and the involvement of a large research team, large-scale validation of the raters’ assessments of actual speech samples has not been systematically carried out. Consequently, questions remain regarding the reliability and consistency of these human evaluations. To address this gap, the present study investigates how widely used temporal fluency features relate to human raters’ evaluations of pronunciation proficiency, while considering potential effects of speaker gender and script complexity. This approach aims to provide empirical insight into the relationship between acoustic–prosodic correlates and subjective proficiency ratings, thereby contributing to a more comprehensive understanding of speech evaluation mechanisms in L2 English learning.

Fluency measures are intrinsically linked to proficiency scoring, serving as key indicators of a learner’s communicative competence. Proficiency encompasses a broad spectrum of linguistic abilities, including grammatical accuracy, vocabulary range, and overall communicative effectiveness (Huensch & Tracy-Ventura, 2017). Fluency, often defined as the smoothness and temporal efficacy of speech production, includes metrics such as speech rate, pause frequency and duration, and repair mechanisms like self-corrections. Since the 1970s, research interest in fluency has intensified (Fillmore, 1979; Segalowitz, 2010), with ongoing efforts to develop effective assessment methods for fluent pronunciation and speaking in English as a second language (Tavakoli & Wright, 2020). Studies have demonstrated that specific fluency measures, such as articulation rate (speech rate excluding pauses) and mean length of pauses, are strong predictors of overall proficiency in second language learners (Baker-Smemoe et al., 2014). Related work likewise emphasizes that articulation rate and pausing behavior jointly shape perceived L2 fluency across tasks, including read speech (Bosker et al., 2013; Cucchiarini et al., 2002; N. De Jong & Perfetti, 2011; Kallio et al., 2022).

Despite the importance of these acoustic-prosodic correlates, traditional assessments often rely heavily on human raters, whose evaluations can be subjective and inconsistent (N. H. De Jong et al., 2015). Understanding how specific speech characteristics influence raters’ judgments is crucial for developing more objective and reliable assessment tools. Moreover, incorporating these insights into AI systems can enhance their ability to evaluate spoken language proficiency accurately.

A substantial body of research in L2 speech acquisition has shown that pronunciation outcomes are strongly shaped by individual differences (IDs) related to learners’ linguistic experience, cognitive resources, and affective dispositions (Carver, 1990). These include age of acquisition and age of first intensive exposure, the amount and quality of L2 input and use, length and context of residence, motivational and anxiety-related factors, language-learning aptitude and phonological processing skills, as well as task-specific strategic behavior (Piske et al., 2001; Segalowitz, 2010; Kormos, 2014; Suzukida & Saito, 2021). Importantly, such factors can systematically influence both temporal organization (e.g., speech rate and pausing) and segmental outcomes, and they may interact with task demands and rater expectations. Consequently, group-level differences (e.g., gender) and acoustic predictors should be interpreted within this broader individual-differences framework rather than as direct evidence of causal mechanisms (Moyer, 2016).

At the same time, large-scale assessment corpora often prioritize standardized elicitation and acoustic annotation over detailed learner background profiling. The present NIA-funded corpus provides extensive scripted L2 English speech data with temporal and phonetic measurements, but it does not contain direct measures of several key individual-difference constructs (e.g., age of onset of English learning, detailed exposure/use histories, motivation/anxiety questionnaires, or standardized aptitude and phonological awareness measures). We therefore do not aim to offer a comprehensive causal account of pronunciation development. Instead, we examine how temporal speech characteristics relate to ordinal pronunciation accuracy ratings in a controlled assessment setting, while controlling for the learner-level variables available in the corpus (chronological age and proficiency band) and modeling unobserved heterogeneity through random intercepts for speakers and scripts. In this context, any gender-related differences are reported as descriptive associations and interpreted cautiously.

Accordingly, the present study examines how fluency-related attributes—specifically articulation rate and mean silence duration—affect raters’ pronunciation proficiency scores for utterances produced by Korean learners of English at varying proficiency levels. Leveraging the extensive NIA-funded corpus enables a fine-grained analysis of the relationship between measurable acoustic features and subjective human ratings. The findings are expected to inform the development of data-driven AI algorithms for automatic proficiency assessment and deepen our understanding of the cognitive and temporal mechanisms underlying L2 speech production.

Given that both phonetic and fluency measures reflect the underlying cognitive and articulatory mechanisms of speech production, it is essential to ground our analysis in a theoretical model that explains how linguistic planning and articulation interact in real time.

We adopt Levelt’s model of speech production as the theoretical framework for our analysis (Levelt, 1989, 1999). Levelt’s model delineates speech production into three stages: conceptualization, formulation, and articulation. While originally developed for first-language speakers, this model has significant implications for second language (L2) learning and assessment (Skehan, 2009; Tavakoli & Wright, 2020). In L2 speakers, fluency reflects the degree of automatization in language processing, where higher proficiency is associated with more efficient speech production mechanisms, resulting in faster articulation rates and shorter pauses.

Furthermore, we consider the influence of script complexity on pronunciation proficiency. Complex scripts may impose a higher cognitive load on learners, potentially affecting their fluency and articulation (Carver, 1998; Liu, 2011). Assessors might perceive successful navigation of complex scripts as indicative of higher proficiency, which could lead to higher pronunciation scores. Additionally, gender differences in language acquisition have been documented, with some studies suggesting that female learners may exhibit superior pronunciation accuracy compared to males (Byrd, 1992; Erdiana et al., 2019; Moyer, 2016; Samuelsson, 2006; Whiteside, 1995). However, reported gender-related patterns in L2 pronunciation and related abilities vary across populations and tasks, suggesting that both aptitude-related and sociocultural factors may contribute (Es-skare et al., 2018; Reiterer et al., 2013; Wucherer & Reiterer, 2018). Learner attitudes toward technology-mediated oral-skill development may also differ across contexts, which could indirectly shape practice opportunities (Harb et al., 2014). These differences could manifest in variations in speech rate and pausing patterns, influencing overall assessments of proficiency.

However, the relationship between articulation rate and pronunciation proficiency on the one hand, and silence duration and pronunciation proficiency on the other hand, is complex. Habitual speaking rate also varies systematically by speaker characteristics (e.g., age and gender), underscoring the need to interpret rate-based measures as partly speaker-specific (Tsao & Weismer, 1997; Jacewicz et al., 2010). Moreover, increased speed can co-occur with articulatory reduction and changes in segmental clarity, which may differentially affect perceived pronunciation accuracy (Tomaschek et al., 2018; Whitfield & Goberman, 2017). A higher articulation rate might be expected to correlate with higher proficiency due to increased fluency. It is, however, possible even for highly proficient speakers to have a slower articulation rate. Articulation rate can vary widely among individuals, depending on several factors such as personal speaking style, context and formality, communication goals, age, and health. Conversely, a higher silence mean could potentially reflect more deliberate speech patterns, which may either enhance or impede clarity based on the context and the listener’s expectations. Long silences might serve to highlight structural boundaries in speech or introduce dramatic pauses for effect. These longer pauses can also indicate a speaker’s uncertainty or the need for cognitive processing time. Accordingly, the perceptual impact of pauses depends on both duration and placement, and pauses can serve discourse-planning functions in otherwise fluent speech (Campione & Véronis, 2002; Préfontaine, 2013; Préfontaine et al., 2016).

Based on these considerations, we formulate the following research questions:

1. How do articulation rate and mean silence duration influence human raters’ pronunciation proficiency scores for Korean learners of English?
2. Does script complexity affect pronunciation proficiency scores, and is this effect mediated by phonetic features such as articulation rate and mean silence duration?
3. Are there gender differences in pronunciation proficiency scores among Korean learners of English, and how do these differences relate to articulation rate and mean silence duration?

To address these questions, we analyze the substantial dataset constructed through the NIA’s AI training data support program, comprising 400 h of speech from 882 Korean learners of English. Using Cumulative Link Mixed Models (CLMM) (Christensen, 2018, 2019), we model the ordinal pronunciation proficiency scores as a function of the phonetic features, script difficulty levels, and gender, while accounting for individual variability among speakers. CLMMs extend standard ordinal regression for ordered categorical outcomes to clustered data via random effects, which is appropriate for rater-based score data with speaker- and item-level dependencies (Agresti, 2013; Hedeker & Gibbons, 1994).

By elucidating the relationships between these variables, our study seeks to enhance the understanding of factors that influence human raters’ evaluations of pronunciation proficiency. The findings have implications for the development of AI-driven language learning tools, suggesting that incorporating fluency measures and script complexity into automated scoring systems can improve their accuracy and reliability. Additionally, recognizing gender-related differences can inform more personalized approaches to language instruction and assessment.

In summary, this research contributes to the field of second language acquisition by providing empirical evidence on how specific phonetic features and learner characteristics impact pronunciation proficiency assessments. It underscores the limitations of current AI models like ChatGPT in evaluating spoken language fluency due to their inability to process acoustic features, highlighting the importance of developing specialized AI systems for spoken language assessment. The utilization of the NIA-funded speech corpus not only adds robustness to our analysis but also exemplifies the value of large, expertly curated datasets in advancing AI capabilities. The insights gained from this study can inform better teaching methods and assessment criteria, ultimately enhancing the effectiveness of language learning and evaluation.

## 2. Materials and Methods

### 2.1. Participants

This study utilized a large-scale corpus of L2 English speech produced by Korean learners of English. The data were collected as part of a government-funded initiative aimed at developing an AI-assisted speech communication system (Han et al., 2024). A total of 882 participants contributed to the corpus, representing diverse backgrounds in terms of gender, age, and English proficiency levels. However, since the present analysis specifically focuses on gender and proficiency effects, only these two variables were included in the statistical modeling. Detailed descriptions of the corpus construction and annotation procedures are available in Han et al. (2024). Table 1 summarizes the demographic distribution of participants by gender and proficiency level.

Participants’ proficiency levels were defined during corpus construction using CEFR-guided, pragmatic criteria rather than a single standardized placement test score (Han et al., 2024). Specifically, university participants were stratified by academic stage (years 1–2 as beginner, years 3–4 as intermediate, and postgraduates/professional interpreters as advanced). Non-university participants were classified based on study duration in accredited language programs and/or length of overseas residency. Gender was recorded as male or female. Age (13–65 years in the present dataset) was included as a covariate (z-scored) in the statistical models to partially account for individual differences.

### 2.2. Speech Corpus

The speech corpus comprised 400 h of recorded English speech, totaling 114,494 individual utterances. Each participant contributed approximately 0.48 h of speech on average. The overall speech characteristics are summarized in Table 2.

Recordings were collected via a web-based authoring platform optimized for desktop use (Chrome), with participants instructed to record in quiet environments (e.g., private spaces or noise-free classrooms) and earphone use recommended to reduce ambient noise (Han et al., 2024). Device information was recorded as technical metadata. Each recording underwent noise-cancelation processing, and a brief silent padding was inserted at the beginning and end of each segment prior to labeling to prevent onset truncation in browser-based recording (Han et al., 2024). Again, for additional details regarding corpus design and recording specifications, refer to Han et al. (2024). For acoustic feature extraction in the present study, we removed leading and trailing padding silences before computing silence-based measures.

### 2.3. Reading Materials

To examine the influence of script difficulty on pronunciation accuracy, two distinct reading tasks were designed: single-sentence reading and paragraph reading. The reading materials were carefully selected and categorized according to the Common European Framework of Reference for Languages (CEFR) to ensure systematic variation in lexical and grammatical complexity.

Scripts were classified into three levels of difficulty:

- Low Difficulty (Pre-A1 to A1): Contained basic vocabulary and simple grammatical structures suitable for beginner learners.
- Medium Difficulty (A2 to B1): Included more varied vocabulary and moderately complex grammar, appropriate for intermediate learners.
- High Difficulty (B2 to C2): Featured advanced vocabulary and complex grammatical constructs, challenging for advanced learners.

Each participant completed reading tasks that incorporated an equitable distribution of scripts across all three difficulty levels. This design ensured that every speaker engaged with texts of varying complexity, thereby enabling a comprehensive examination of how script difficulty influences pronunciation proficiency and fluency measures.

### 2.4. Procedures

#### 2.4.1. Scoring Rubrics

To ensure consistency in subjective evaluations, the assessment procedure followed established evaluation metrics focusing on pronunciation performance in two complementary dimensions: pronunciation accuracy and prosodic fluency. Pronunciation accuracy was assessed for clarity of segmental phonemes and intelligibility (Klebanov and Madnani, 2022; Zhang et al., 2021), while prosodic fluency captured stress, rhythm, intonation, speaking rate, and pause behavior. This framing is consistent with the established distinction among foreign accent, comprehensibility, and intelligibility in L2 speech assessment (Munro & Derwing, 1998; Loukina et al., 2015). The scoring rubrics are presented in Table 3.

#### 2.4.2. Assessor’s Scoring Process

Assessors evaluated each sample using a custom web-based interface, assigning scores from 1 to 5 based on pronunciation accuracy. For the English subset analyzed here, the assessor panels consisted of native speakers of English selected under stringent criteria (e.g., PhD-level faculty with relevant teaching experience or professional interpreters), and assessors underwent rubric training and calibration prior to full-scale scoring (Han et al., 2024). A dual-assessor system ensured rigorous evaluation; each utterance was independently rated by two assessors, and substantial discrepancies were adjudicated by a third expert. The composition of the assessor panel is shown in Table 4.

To assess inter-rater reliability, Krippendorff’s alpha (α) was employed (De Swert, 2012; Marzi et al., 2024). In large-scale linguistic assessments, α values of 0.60 or higher are generally considered acceptable. In the present study, an average α ≈ 0.65 indicated a substantial level of agreement across assessors, reflecting reliable pronunciation evaluations despite the large number of samples and raters.

### 2.5. Feature Extraction

Pronunciation accuracy scores were analyzed in relation to two acoustic–phonetic features: articulation rate (AR) and mean silence duration (SilMean), as in Table 5. AR indexes speech speed excluding silent intervals, and SilMean indexes the average duration of within-utterance silence intervals. Because the corpus preprocessing adds brief silent padding at segment boundaries (Han et al., 2024), we trimmed leading and trailing padding silences before computing silence-based measures so that SilMean reflects internal (within-utterance) silences only.

Based on prior research, we test whether gender is associated with pronunciation accuracy ratings. For linguistic complexity of the reading scripts, we do not posit a single directional hypothesis a priori; greater linguistic complexity may increase cognitive load and hinder performance, but it may also affect rater judgments via task/context effects (e.g., raters inferring higher proficiency from more complex materials). Accordingly, we examine its association with ratings while interpreting any effect cautiously as task-dependent.

### 2.6. Analysis

**Figure 1:**
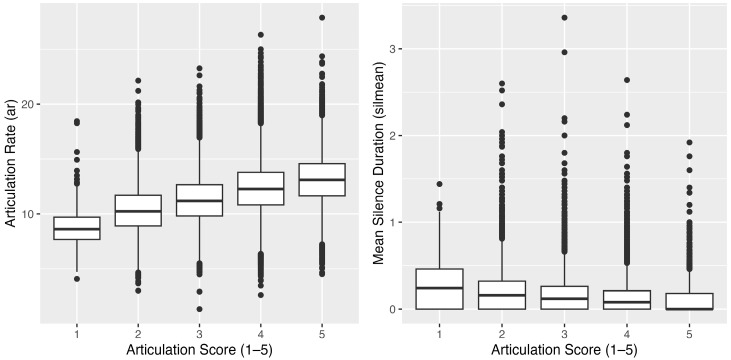
Box plots of articulation score against phonetic features.

To gain insight into how temporal-phonetic measures pattern across human ratings, we first report descriptive statistics for articulation rate and mean silence duration (Table 6) and visualize their relationships with articulation ratings using box plots (Figure 1).

In conjunction with the descriptive statistics, Figure 1 provides a visual exploration of the data. The figure comprises a series of box plots that depict the distribution of articulation clarity scores against two key speech characteristics: articulation rate and mean silence duration.

The vertical axis of the box plots represents articulation score, scored on a 5-point scale where 1 corresponds to low clarity and 5 denotes high clarity. Two distinct box plot series are displayed. The first series plots articulation rate on the horizontal axis, illustrating the distribution of articulation scores at varying speeds of speech. The articulation rate is measured in phonemes per second, providing an indication of the speaker’s fluency. The second series align mean silence duration on the horizontal axis, showcasing how longer silence intervals within speech correlate with the perceived clarity of pronunciation. These box plots allow for a comparative analysis of how different articulation rates and silence durations are associated with pronunciation clarity scores. A higher articulation rate might be expected to correlate with higher clarity due to increased fluency. Conversely, a higher silence mean could potentially reflect more deliberate speech patterns, which may either enhance or impede clarity based on the context and the listener’s expectations. By interpreting these plots, we aim to discern patterns that could inform pedagogical approaches and automated assessment algorithms in L2 English learning, focusing on how distinct speech characteristics influence pronunciation accuracy.

Figure 1 reveals a connection between articulation rate and perceived clarity of pronunciation. While a faster articulation rate is commonly associated with fluency and often signals higher proficiency in language, its direct impact on clarity is not straightforward. The distribution of clarity scores across various articulation rates underscores that rapid speech does not invariably result in clearer communication. Indeed, speech clarity is also influenced by other factors such as precise enunciation, appropriate stress, controlled rhythm, and correct intonation. Each of these plays a pivotal role in the perception and comprehension of spoken language.

Regarding the influence of silence on speech fluency, our data suggest that shorter silence durations—reflected in the ‘silmean’ metric—often correspond with a more fluid and rapid delivery, which might be interpreted as confident and proficient language use. However, the relationship between silence duration and speech clarity is complex. Long silences, for example, might serve to highlight structural boundaries in speech or introduce dramatic pauses for effect. These longer pauses can also indicate a speaker’s uncertainty or the need for cognitive processing time. The distribution of articulation clarity scores in relation to silence mean values reveals that silence plays an integral communicative role, with implications for both the production and assessment of fluent speech.

This variability in articulation clarity against articulation rate and silence mean highlights the multifaceted attributes of speech. Assessing language proficiency, particularly pronunciation clarity, therefore requires a comprehensive consideration of multiple phonetic factors. It is not sufficient to evaluate speech based solely on the speed of delivery; one must also consider the strategic use of silence and other prosodic features to fully grasp a speaker’s command of language.

Given the complexity observed in the data, our next step is to develop a statistical model that can accurately predict articulation clarity scores. To achieve this, we will be employing a statistical model that incorporates factors such as gender and script difficulty level, alongside phonetic features like articulation rate and silence mean. This approach will allow us to better understand the relative impact of these variables on language proficiency and provide a more nuanced evaluation of pronunciation clarity beyond mere speech velocity, encompassing the thoughtful employment of pauses and the interplay of various prosodic elements essential for capturing the essence of a speaker’s linguistic ability.

#### Modeling of Ordered Pronunciation Scoring

Previous methodological work cautions against treating ordinal ratings as interval-scale data because doing so can distort inference—for example, by reducing statistical power and potentially inflating error rates (e.g., false positives/Type I errors) under common analysis assumptions (Liddell & Kruschke, 2018; Howcroft & Rieser, 2021). Following these recommendations, we model pronunciation ratings using Cumulative Link Mixed Models (CLMM), which are specifically designed for ordered categorical outcomes.

The statistical model ‘clmm’, short for Cumulative Link Mixed Models, is expressly designed for data where the response variable is ordinal, such as rating scales or Likert-type items (Christensen, 2018, 2019)^1^. This function is apt for our needs, as it accommodates the discrete nature and inherent order of ordinal categories, permitting the application of ordinal logistic regression with one or more random effects. The package also allows for the inclusion of both fixed effects, which represent the systematic influences on the response variable that we wish to study, and random effects, which account for random variations that may arise from grouped data or repeated measurements. Therefore, in this paper, we employ ‘clmm’ to finely tune our analysis to the subtleties of ordinal response data, facilitating a more precise and theoretically sound statistical modeling.

As such, we applied ordered logistic regression to the data. Ordered logistic regression is a case of the multinomial logit model in which the categories are ordered. The Ordinal Logistic regression model, also known as the ordinal logit model, employs the logistic function to model the relationship between probabilities and log-odds. The fundamental form of the model is expressed as

$$logit(P(Y\leq j))=\alpha_{j}-(\beta_{1}{Χ}_{1}+\beta_{2}{Χ}_{2}+\ldots +\beta_{k}{Χ}_{k})$$

Here P(Y≤j) denotes the probability that the dependent variable *Y* assumes a value less than or equal to a certain ordinal level *j*. In the context of CLMMs, we consider a scenario where we have ‘*J*’ ordered categories for the response variable. For any given individual ‘*i*’ with an ordinal response ‘*Y_i_*’, the probability that this response is exactly ‘*j*’ is denoted as $p_{ij}=P\left(Y_{i}=j\right)$ for ‘*j*’ = 1, …, ‘*J*’. However, since we are dealing with ordinal data, we focus on cumulative probabilities. That is, for each individual ‘*i*’ and category ‘*j*’, we compute $\gamma_{ij}=P\left(Y_{i}\leq j\right)$, which is the probability that the individual’s response falls at or below a certain ordinal level ‘*j*’. A key characteristic of these cumulative probabilities is that they are non-decreasing as ‘*j*’ increases. This is because, as we move to higher ordinal categories, the cumulative probability naturally encompasses all the lower categories. Furthermore, these probabilities are invariant under the combination of adjacent categories due to their cumulative nature.

The *α_j_* represents the threshold or cutpoint for each ordinal category *j*, and *β_i_* are the coefficients for the independent explanatory or predictor variables. The *α_j_* parameters are estimated typically using Maximum Likelihood Estimation (MLE). The values of *α_j_* are found by maximizing the likelihood function based on the observed data. They define the boundaries between the ordinal response categories on the underlying latent scale. The “ordinal” package in R was used for this purpose (Christensen, 2018, 2019).

Upon selecting cumulative link mixed models (CLMMs; R package ordinal (Version 2023.12-4.1), we modeled 5-category articulatory accuracy ratings (artScore5) using a logit link. To address concerns about uncontrolled proficiency and task effects, we included learner proficiency (ability) and age as covariates, and modeled both speaker- and script-level variability via random intercepts for SpeakerID and scriptId.

- Formula: artScore_5 ~ lingComplexity + ability + gender + age_z + testType + ar_z + (1 | SpeakerID) + (1 | scriptId).
- Formula: artScore_5 ~ lingComplexity + ability + gender + age_z + testType + silmean_z + (1 | SpeakerID) + (1 | scriptId).
- Formula: artScore_5 ~ lingComplexity + ability + gender + age_z + testType + ar_z + silmean_z + (1 | SpeakerID) + (1 | scriptId).
- Formula (exploratory): artScore_5 ~ lingComplexity + ability + gender + age_z + testType + silmean_z * ability + (1 | SpeakerID) + (1 | scriptId).

All continuous predictors (age, articulation rate, and mean silence duration) were standardized (z-scored) to improve numerical stability and facilitate effect-size comparisons. SpeakerID captures speaker-specific baseline tendencies in ratings (random intercept), whereas scriptId captures item/task-related differences that may influence ratings independent of the speaker.

We fitted (i) an AR-only model, (ii) a SilMean-only model, and (iii) a combined model including both temporal predictors. Model fit was compared using AIC. In addition, we ran exploratory interaction models to test whether temporal effects vary by proficiency (ability). Finally, to examine construct alignment, we repeated the analysis using prosody scores (prosScore_5) as an outcome, because temporal cues are theoretically more central to prosodic fluency than to segmental articulation accuracy.

Finally, to aid interpretability, we focus on P(score ≥ 4) as a practically meaningful “high-score” criterion in the main text. Because the CLMM specifies the full ordinal outcome distribution, complementary probability targets (e.g., P(score ≥ 3) and P(score = 5)) are straightforwardly derived from the same fitted model.

## 3. Results

### 3.1. Descriptive Overview

Across the full corpus, articulation accuracy ratings (artScore_5) were distributed as follows: 1 (*n* = 464), 2 (*n* = 14,909), 3 (*n* = 17,428), 4 (*n* = 62,881), and 5 (*n* = 18,812). Prosody ratings (prosScore_5) showed a similar distribution: 1 (*n* = 600), 2 (*n* = 15,104), 3 (*n* = 15,664), 4 (*n* = 66,087), and 5 (*n* = 17,039).

For the CLMM analyses reported below, we used listwise-complete observations for the outcome and predictors. This yielded *n* = 114,494 utterances from 882 speakers (SpeakerID). All models included random intercepts for both speakers and scripts (scriptId) to account for speaker-level and item/task-level dependencies in the ratings.

### 3.2. Articulation Accuracy Outcome (Main Analysis)

We compared CLMMs that included (i) articulation rate only (m_ar), (ii) mean silence duration only (m_sil), (iii) both temporal predictors jointly (m_both), and (iv) exploratory proficiency-interaction variants. As shown in Table 7, the combined model (m_both) provided the best fit (lowest AIC), indicating that articulation rate and mean silence duration contribute complementary information in predicting human ratings.

Fixed effects from the best-fitting combined model (m_both) are reported in Table 8. In this model, a higher articulation rate was associated with higher articulation ratings, whereas a longer mean silence duration was associated with lower ratings, even after controlling for linguistic complexity, proficiency (ability), gender, age, and test type. Speaker-level variance was substantially larger than script-level variance, supporting the importance of modeling between-speaker differences. Notably, the fixed effect of the corpus proficiency band (ability) was small and not statistically reliable in the additive model (Table 8 and Table 9), a pattern we discuss further in the Limitations.

### 3.3. Proficiency-Dependent Temporal Effects (Exploratory)

To evaluate whether temporal cues relate to ratings differently across proficiency levels, we fitted interaction models. For mean silence duration, the interaction between proficiency (ability) and silmean_z was significant (ability × silmean_z estimate = 0.0635, *p* = 5.22 × 10^−16^), suggesting that longer silences are more strongly penalized for lower-proficiency speakers. However, the interaction model did not improve overall fit relative to the additive models (Table 7), so we interpret this moderation pattern as exploratory.

### 3.4. Prosody Outcome (Construct-Alignment Analysis)

Because temporal cues are theoretically more central to prosodic fluency than to segmental articulation accuracy, we repeated the CLMM analysis using prosody ratings (prosScore_5) as the outcome. Table 9 shows that both articulation rate and mean silence duration were strongly associated with prosody ratings; notably, the magnitude of the AR and SilMean coefficients was larger than in the articulation-accuracy model, supporting construct alignment.

### 3.5. Predicted Probabilities

To facilitate interpretation of the joint CLMM with articulation rate and mean silence duration (m_both), we additionally visualized (i) the estimated cutpoints (thresholds) that separate adjacent score categories, and (ii) model-implied probabilities under representative covariate settings. Unless otherwise stated, probabilities are marginal (fixed-effect) predictions with random effects set to zero and other continuous predictors held at their mean (z = 0), while categorical predictors are shown across their observed levels.

**Figure 2:**
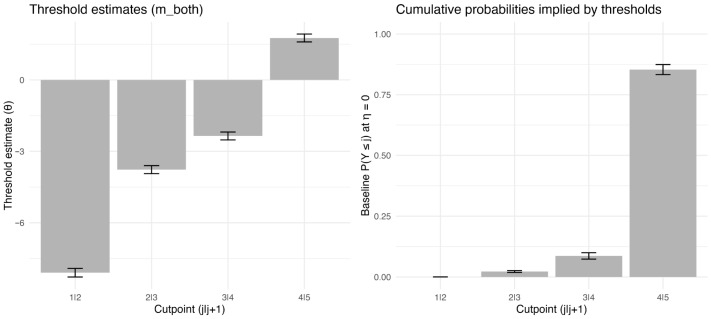
Estimated cutpoints (thresholds) from the joint CLMM (m_both) and the baseline cumulative probabilities implied by these cutpoints. For a 5-point ordinal outcome, four cutpoints (1|2, 2|3, 3|4, 4|5) partition the latent propensity scale into five ordered categories; baseline cumulative probabilities are shown at η = 0 (all predictors at reference/mean levels).

As shown in Figure 2, the cutpoints increase monotonically, reflecting the progressively stricter latent criteria required to obtain higher ratings. In particular, the highest cutpoint (4|5) is separated from the lower cutpoints, consistent with a ceiling tendency in which the maximum score requires substantially stronger evidence on the latent pronunciation-quality continuum than moving between intermediate scores.

### 3.6. Model-Implied Cutpoints and Extended Predicted Probability Visualizations

**Figure 3:**
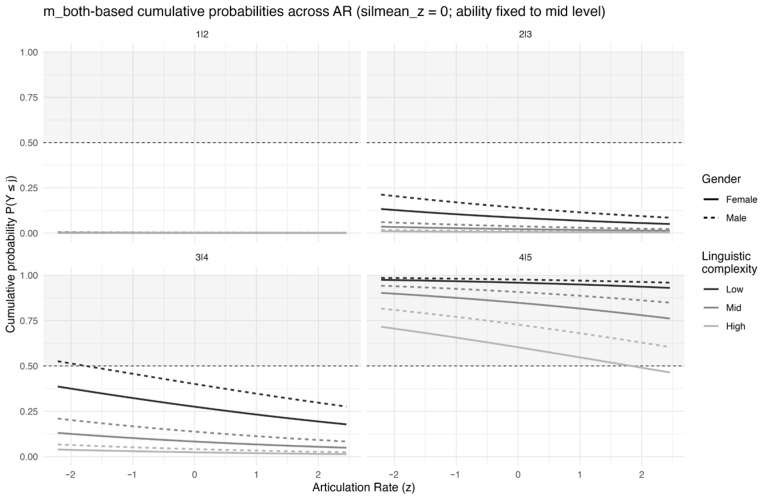
Model-implied cumulative probabilities P(Y ≤ j) across articulation rate (z) for each cutpoint (1|2, 2|3, 3|4, 4|5) based on m_both. Curves illustrate how increases in articulation rate shift cumulative probability mass toward higher score categories, with other predictors held constant.

Figure 3 decomposes the ordinal prediction into cutpoint-specific cumulative curves, which clarifies where in the scale articulation rate exerts the largest shifts. Across conditions, changes in articulation rate are most visible around the upper cutpoints (3|4 and 4|5), indicating that temporal fluency is particularly informative for distinguishing higher ratings rather than merely separating low from medium scores.

**Figure 4:**
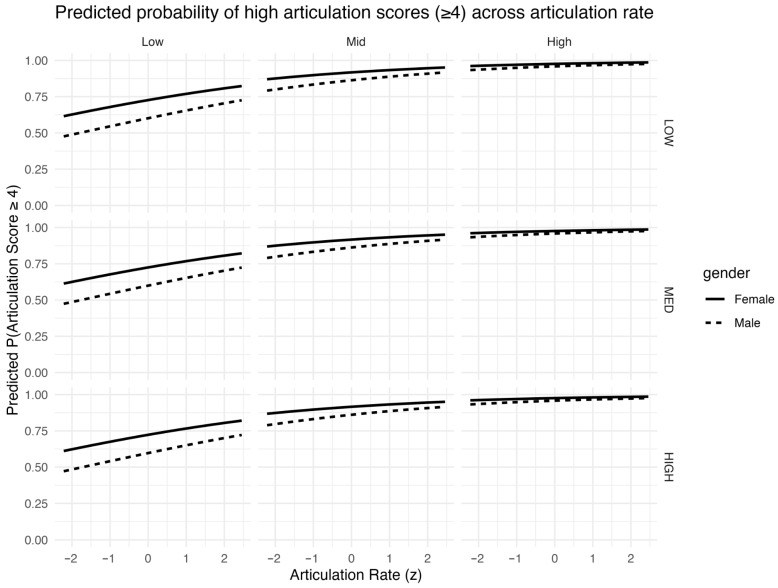
Predicted probability of receiving a high articulation score (P(score ≥ 4)) as a function of articulation rate (z), derived from m_both with mean silence duration fixed at its mean (silmean_z = 0). Panels are stratified by linguistic complexity (columns) and proficiency group (rows); line type indicates gender. The *y*-axis spans the full probability scale (0–1).

To aid interpretability, we visualized marginal model predictions as the probability of receiving a high articulation rating (score ≥ 4) across the observed range of articulation rate. Probabilities were computed from the fitted CLMM using fixed effects only (random effects set to zero) with age held at its mean (age_z = 0) and testType set to the reference level, while mean silence duration was fixed at its mean (silmean_z = 0). Consistent with the positive fixed effect of articulation rate in the joint model, Figure 4 shows a monotonic increase in the probability of receiving a high articulation rating (P(score ≥ 4)) as articulation rate increases, with steeper gains in strata where baseline probabilities are lower (e.g., lower proficiency and lower linguistic complexity). On the full 0–1 scale, the figure also makes clear that some strata—particularly higher-proficiency and higher-complexity conditions—approach ceiling across much of the observed AR range, whereas lower-proficiency/low-complexity strata retain a wider dynamic range and exhibit more pronounced increases in P(score ≥ 4) as articulation rate rises.

**Figure 5:**
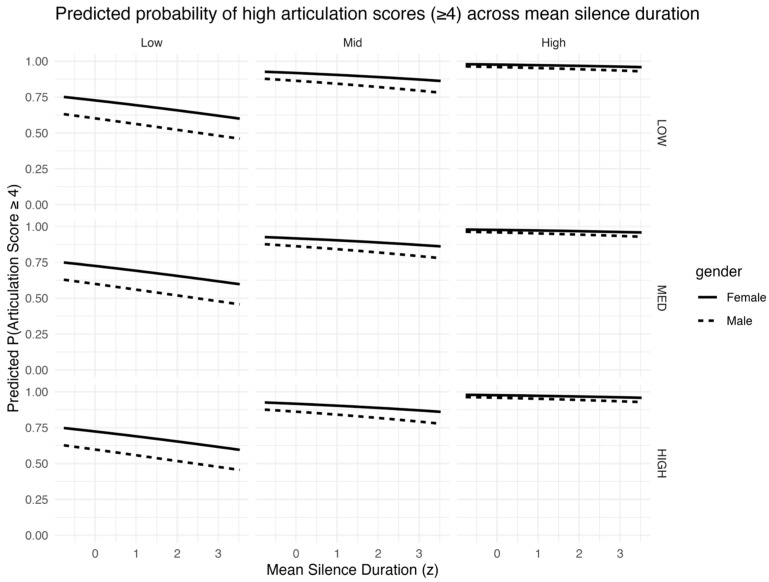
Predicted probability of receiving a high articulation score (P(score ≥ 4)) as a function of mean silence duration (z), derived from m_both with articulation rate held at its mean (ar_z = 0). Panels are stratified by linguistic complexity (columns) and proficiency group (rows); line type indicates gender.

Figure 5 complements the articulation-rate visualization by isolating the role of pausing behavior; holding articulation rate constant (ar_z = 0), the predicted probability of a high rating decreases as mean silence duration increases, indicating that longer within-utterance silences are associated with lower articulation ratings even after accounting for articulation rate.

**Figure 6:**
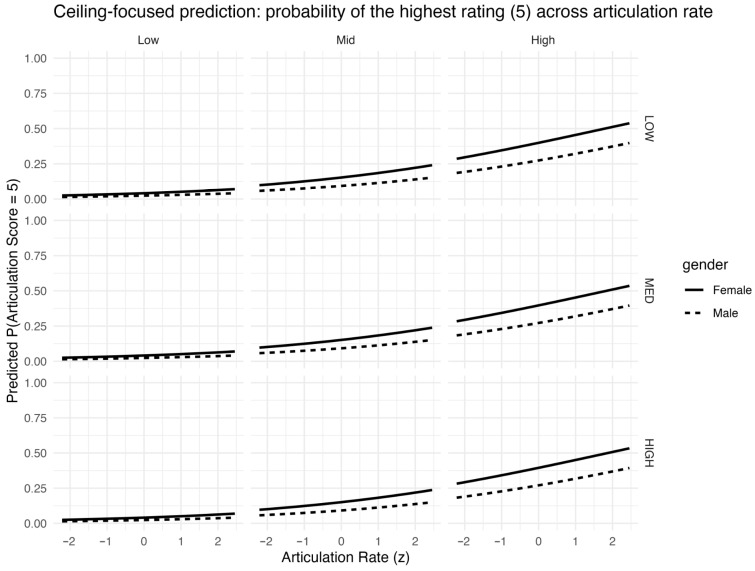
Predicted ceiling probability P(score = 5) as a function of articulation rate (z), derived from m_both with mean silence duration fixed at its mean (silmean_z = 0). Panels are stratified by linguistic complexity (columns) and proficiency group (rows); line type indicates gender.

Focusing on the top category, Figure 6 highlights the ceiling dynamics; while a higher articulation rate increases the probability of achieving the maximum score, the increase tends to plateau under conditions where baseline performance is already high, consistent with the large separation implied by the 4|5 cutpoint.

**Figure 7:**
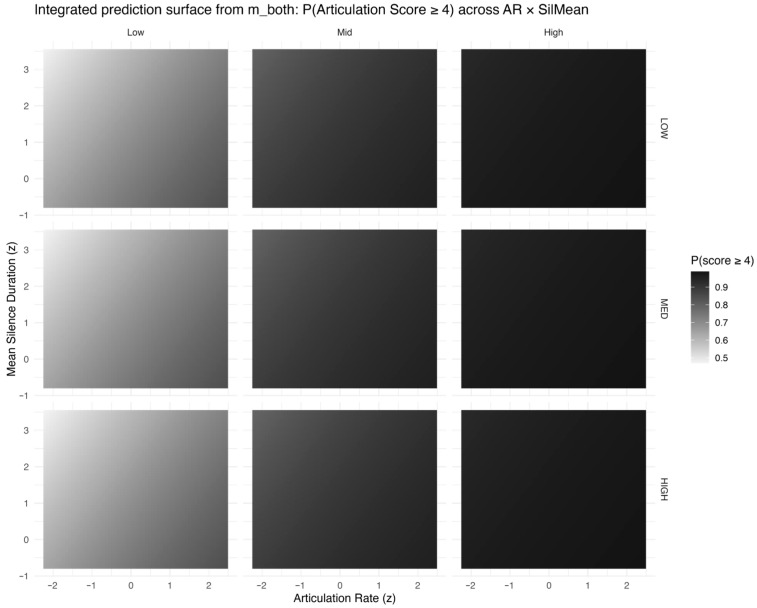
Integrated heatmap of the predicted probability of receiving a high articulation score (P(score ≥ 4)) over the joint space of articulation rate (z) and mean silence duration (z), derived from m_both. Darker shades indicate higher predicted probabilities. Panels are stratified by linguistic complexity (columns) and proficiency group (rows).

The joint surface in Figure 7 summarizes the combined temporal profile; the highest probabilities of high articulation scores occur in regions with faster articulation rate and shorter mean silence duration, whereas slower articulation coupled with longer silences yields markedly lower predicted probabilities. This integrated view supports treating articulation rate and pause structure as complementary temporal correlates of rated pronunciation performance.

Overall, the results show that articulation rate and mean silence duration provide complementary information about human pronunciation ratings in read L2 speech; the joint model (m_both) fit the data best, and both temporal predictors retained strong associations with scores even after controlling for proficiency, linguistic complexity, age, gender, and test type, with speaker- and script-level dependencies modeled via random intercepts. The predicted-probability visualizations (Figure 4, Figure 5 and Figure 6) further illustrate how these effects manifest across learner groups and task conditions and highlight ceiling behavior at the top end of the rating scale. In the Discussion, we interpret these patterns in relation to the study’s research questions and consider their implications for construct-aligned human and AI-supported pronunciation assessment.

## 4. Discussion

### 4.1. RQ1: Temporal-Phonetic Predictors and Pronunciation Ratings

Across models, faster articulation (higher AR) and shorter silent intervals (lower SilMean) were consistently associated with higher human ratings. Crucially, these relationships remained robust after controlling for proficiency (ability), age, gender, and test type, and after modeling speaker and script variability via random intercepts. This supports the interpretation that the temporal organization of speech provides listeners with salient cues that correlate with perceived pronunciation quality in read L2 speech.

At the same time, the construct-alignment analysis indicates that temporal cues are even more predictive of prosody ratings than of articulation ratings (Table 9 vs. Table 8). This pattern is consistent with the view that listeners use fluency-related timing cues when judging speech, and it reduces (but does not eliminate) the concern that temporal predictors are completely misaligned with the rating rubric. We therefore characterize the outcome as human pronunciation ratings (articulation clarity vs. prosody) rather than a broad ‘pronunciation proficiency’ construct.

### 4.2. RQ2: Linguistic Complexity and Task-Related Effects

Linguistic complexity showed a significant positive linear trend in both outcomes. Because script difficulty is not expected to directly improve segmental accuracy, we interpret this effect cautiously as reflecting task-related differences and/or rater expectations rather than a causal impact of complexity on pronunciation. Importantly, including scriptId as a random intercept directly addresses the concern that item/task effects could inflate fixed effects; nevertheless, the remaining fixed complexity trend suggests that future work should further disentangle textual complexity from rater bias (e.g., via counterbalanced designs or rater-blind complexity manipulations).

### 4.3. RQ3: Gender Differences and Individual Variability

Male speakers received lower ratings on average in both articulation and prosody models (Table 8 and Table 9). We interpret this association cautiously. Even though we controlled for proficiency category and age and modeled large speaker-level variability, the corpus does not include other well-known individual-difference predictors (e.g., age of acquisition, amount of L2 exposure, motivation, aptitude, or phonological awareness). Accordingly, we avoid strong causal claims and frame gender effects as descriptive associations that warrant targeted follow-up studies with richer learner background measures.

### 4.4. Implications for AI-Supported Pronunciation Assessment

From an applied perspective, the results highlight that timing-derived measures can support automated feedback or scoring, but only when the target construct is defined clearly. For segmental-focused scoring, temporal cues may enter indirectly through listeners’ global impressions; for prosody/fluency constructs, temporal measures provide more direct evidence. Future AI-based systems should therefore (i) separate segmental and suprasegmental targets, (ii) include item and speaker controls, and (iii) evaluate fairness across learner subgroups.

## 5. Limitations and Future Research Directions

Several limitations should be considered. Firstly, the data are based on scripted read speech, which constrains the range and function of pauses and may not generalize to spontaneous interaction. Secondly, proficiency labels are coarse metadata categories; while we included them as controls, future work should incorporate standardized proficiency measures. Although proficiency is theoretically central to L2 pronunciation, the weak main effect of ability in the present models likely reflects limitations of the available metadata rather than an absence of proficiency-related differences in speech. The corpus proficiency bands were created for pragmatic stratification during corpus construction and may be too coarse to capture meaningful within-band variability. Thirdly, rater-level identifiers were not available for the present analyses, preventing explicit modeling of rater severity/leniency. Fourthly, although we removed leading/trailing padding silences during feature extraction (see also Han et al., 2024), our SilMean measure still averages across pause types and does not distinguish boundary from hesitation pauses. Future work should incorporate pause classification and prosodic boundary detection.

## 6. Conclusions

Using a large L2 English read-speech corpus, we examined how articulation rate and mean silence duration relate to human pronunciation ratings. CLMM analyses controlling for proficiency, age, gender, and test type—and accounting for speaker and script dependencies—showed that faster articulation and shorter silences predict higher articulation ratings. Temporal cues were even more strongly associated with prosody ratings, supporting construct alignment. Although gender differences were statistically detectable, they were small in magnitude compared with the temporal predictors and should be interpreted cautiously given the absence of richer individual-difference measures in the corpus. Taken together, these findings suggest that temporal organization provides robust information about perceived pronunciation quality in read L2 speech, while underscoring the importance of modeling task effects, defining scoring constructs precisely, and interpreting subgroup differences cautiously.

## Figures and Tables

**Table 1:** Demographic characteristics of study participants.

Demographics	Category	Percentage
Speakers	Total	882
Gender	Female	63.95%
	Male	36.06%
Proficiency	High	20%
	Medium	30%
	Low	50%

**Table 2:** Speech characteristics of the corpus.

Speech Characteristics	Value
Duration (hour)	400
Samples	114,494
Avg. Tokens	26.87
Duration/Speaker (h)	0.48

**Table 3:** Scoring rubrics for pronunciation accuracy and prosodic fluency (adapted from Han et al., 2024).

Score	Pronunciation Accuracy	Prosodic Fluency
5	No errors or awkwardness of segmental phonemes in the speech. Easy to understand.	Natural stress, rhythm, and intonation. The speaking rate is moderate, and the number and duration of pauses are natural. There are few speech mistakes, and the pauses are appropriately used to separate units of speech.
4	A few errors or awkwardness of segmental phonemes in the speech. But intelligibility is not significantly affected.	Slightly awkward stress, rhythm, and intonation. The speaking rate is mostly consistent, with some hesitations and breaks. The pauses are appropriately used to separate units of speech, but their number and duration are slightly awkward.
3	Some errors or awkwardness of segmental phonemes in the speech. Intelligibility is somewhat affected due to certain consistent errors.	Somewhat awkward stress, rhythm, and intonation. The speaking rate is inconsistent and a bit slow, with frequent breaks. The pauses are not appropriately used to separate units of speech.
2	Frequent errors or awkwardness of segmental phonemes in the speech. Intelligibility is only achieved when the listener pays attention to the speaker’s intonation due to some persistent pronunciation errors.	Considerably awkward stress, rhythm, and intonation. The speaking rate is slow, with many breaks. The pauses last long and do not appropriately separate units of speech.
1	The speech lacks clarity of segmental phonemes, with too many errors and awkwardness. Hard to understand.	Terrible stress, rhythm, and intonation. The speaking rate is too slow, with too many breaks. The pauses last too long and do not serve to separate units of speech at all.

**Table 4:** Composition of assessment panel.

Assessment Panel	Assessors	28
	Groups of Two	14
	Samples per Group	8178

**Table 5:** Phonetic feature calculations for speech analysis.

Feature	Abbr.	Formula
Articulation Rate	ar	Total number of phonemes/(Total speaking time − Sum of all silent intervals)
Mean Silence Duration	SilMean	Total sum of silence intervals/Number of silence intervals

**Table 6:** Descriptive statistics for phonetic features.

Phonetic Features	Min	Max	Median	Mean	Std. Dev.
Articulation Rate	1.35	27.88	11.98	12.05	2.40
Silence Mean	0	3.36	0.08	0.138	0.180

**Table 7:** Model comparison (AIC) for articulation accuracy CLMMs.

Model	df	AIC
m_ar (AR only)	13	135,832.4
m_sil (SilMean only)	13	135,815.0
m_both (AR + SilMean)	14	135,446.9
m_ar_int (AR × ability)	14	135,792.5
m_sil_int (SilMean × ability)	14	135,796.3

**Table 8:** Fixed effects from the combined CLMM (m_both) predicting articulation accuracy ratings.

Predictor	Estimate	SE	z	*p*	OR
lingComplexity (linear)	1.933	0.622	3.106	0.00189	6.908
lingComplexity (quadratic)	−0.052	0.365	−0.142	0.88689	0.949
ability (linear)	−0.013	0.614	−0.021	0.98331	0.987
gender (Male)	−0.566	0.130	−4.358	1.31 × 10^−5^	0.568
age_z	−0.297	0.057	−5.226	1.73 × 10^−7^	0.743
testType	−0.282	0.020	−14.045	<2 × 10^−16^	0.754
ar_z	0.228	0.012	19.069	<2 × 10^−16^	1.257
silmean_z	−0.163	0.008	−19.426	<2 × 10^−16^	0.850

Random-effects variances (m_both): scriptId = 0.1218 (SD = 0.349); SpeakerID = 1.9794 (SD = 1.407).

**Table 9:** Fixed effects from the CLMM predicting prosody ratings (prosScore_5).

Predictor	Estimate	SE	z	*p*	OR
lingComplexity (linear)	1.899	0.585	3.247	0.00117	6.677
lingComplexity (quadratic)	−0.115	0.344	−0.335	0.73732	0.891
ability (linear)	−0.141	0.577	−0.244	0.80705	0.869
gender (Male)	−0.537	0.123	−4.368	1.26 × 10^−5^	0.585
age_z	−0.274	0.054	−5.094	3.50 × 10^−7^	0.760
testType	−0.234	0.021	−11.360	<2 × 10^−16^	0.792
ar_z	0.409	0.013	32.354	<2 × 10^−16^	1.505
silmean_z	−0.202	0.009	−23.478	<2 × 10^−16^	0.817

Random-effects variances (prosody model): scriptId = 0.1767 (SD = 0.420); SpeakerID = 1.7705 (SD = 1.331).

## Data Availability

This work utilized the dataset titled *Educational English Speech Data by Korean Speakers* (2020) from AI Hub, provided by the National Information Society Agency (NIA), Republic of Korea. The dataset is available for domestic access under approval at https://aihub.or.kr/ (10 March 2024).

## Note

1 The ‘polr’ function from the ‘MASS’ package in R is another commonly used method for fitting ordinal logistic regression models for ordinal categorical data (Venables & Ripley, 2002). However, it lacks the capability to include random effects directly in the model. As a result, ‘polr’ was deemed unsuitable for our analysis that required mixed-effects modeling.
